# Dyadic Coping and Communication as Predictors of 10-Year Relationship Satisfaction Subgroup Trajectories in Stable Romantic Couples

**DOI:** 10.3390/bs15101361

**Published:** 2025-10-05

**Authors:** Michelle Roth, Fridtjof W. Nussbeck, Selina A. Landolt, Mirjam Senn, Thomas N. Bradbury, Katharina Weitkamp, Guy Bodenmann

**Affiliations:** 1 Department of Psychology, Clinical Psychology for Children/Adolescents and Couples/Families, University of Zurich, 8050 Zurich, Switzerland; 2 Department of Psychology, Methods for Intensive Data in Psychology, University of Konstanz, 78464 Konstanz, Germany; 3 Department of Psychology, Clinical Psychology, University of California, Los Angeles, CA 90095, USA

**Keywords:** romantic couples, changes in relationship satisfaction, dyadic coping, communication, subgroup trajectories

## Abstract

Given the importance of relationship satisfaction and the detrimental effects of its decline in romantic couples, it is crucial to understand how relationship satisfaction develops over time in long-term stable relationships and to identify predictors that explain such long-term changes. Building upon previously identified subgroups with distinct trajectories of relationship satisfaction, our objective was to examine whether two types of relationship skills—dyadic coping and communication—predict subgroup trajectories. We followed 300 mixed-gender couples over 10 years in annual assessments and applied Dyadic Latent Class Growth models with predictors. Our results suggest that subgroups of relationship satisfaction trajectories can be differentiated by both baseline levels and changes in relationship skills. Couples with high and relatively stable satisfaction were distinguished from those with declining satisfaction primarily by baseline negative communication (women’s report) and a deterioration in dyadic coping. Couples with the lowest initial satisfaction exhibited the least beneficial relationship skills but increased their satisfaction over time, likely due to observed improvements in their skills. These findings have important public health implications, as modifiable relationship skills can be targeted in prevention, counseling, or therapy to help couples develop and sustain improvements in their relationship skills to protect their relational well-being in the long term.

## 1. Introduction

Forming and maintaining significant, positive, and lasting interpersonal relationships has been described as a fundamental human desire, with romantic relationships being of particular importance (Baumeister & Leary, 1995). Thereby, higher relationship satisfaction in romantic couples is beneficial for various life domains, such as life satisfaction (Be et al., 2013) and psychological well-being (Proulx et al., 2007), including lower distress (Hawkins & Booth, 2005) and depression (Beach et al., 2003), as well as physical health and longevity (Robles et al., 2014). Moreover, it impacts child well-being (Amato, 2000) and can spill over to work–life (Du et al., 2018). Given this importance, it is crucial to understand how relationship satisfaction changes over time and which factors within a romantic relationship contribute to this change. While the emphasis is typically placed on newlywed couples to understand the initial stages of romantic relationships and potentially explain relationship dissolution (e.g., Lavner & Bradbury, 2010), a noteworthy proportion of couples remain stable over prolonged periods of time, as noted by Karney and Bradbury (2020), yet there is a deficiency of knowledge and understanding about the development of the relationship and its predictors within those couples. This becomes particularly evident in a recent meta-analysis on relationship satisfaction in couples, which found that the majority of studies examined couples with a relationship duration of 0–10 years, followed by considerably fewer studies on couples with a duration of 10–20 years, and only a small number of studies on couples with longer relationship durations (Bühler et al., 2021).

### 1.1. Changes in Relationship Satisfaction

Looking at how relationship satisfaction changes over time, earlier longitudinal studies (e.g., D. R. Johnson et al., 1992; Karney & Bradbury, 1997), as well as a recent meta-analysis (Bühler et al., 2021), consistently observed relationship satisfaction to decline over time within a given relationship, with the largest declines in young adulthood and the first years of a relationship. Yet, theoretical perspectives and empirical evidence suggest the possibility of different trajectories of relationship satisfaction over the course of a given relationship. While the gradual disillusionment model (e.g., Huston et al., 2001; Huston & Houts, 1998) or the honeymoon-is-over effect (Kurdek, 1998, 1999) suggest a decline in relationship satisfaction, particularly in the early years of marriage, implications from the attachment theory (Bowlby, 1980; Hazan & Shaver, 1987) would suggest an increase in relationship satisfaction with longer relationship duration due to a more secure attachment (see Bühler et al., 2021 for an overview of theoretical models). Yet, there is evidence that the trajectories are more complex in nature, and not all couples follow this average decline: In a review by Proulx et al. (2017) on studies applying group-based approaches, different subgroups of change in relationship satisfaction over time were identified. The integrated results point to either stability or decline in relationship satisfaction, with minimal evidence for increasing relationship satisfaction over time (Proulx et al., 2017). Compared to most earlier studies (Proulx et al., 2017), more recent studies accounted for the dyadic nature of the data (Kanter et al., 2023; Kanter & Proulx, 2021; Roth et al., 2025; Stadelmann, 2022), allowing for conclusions not only about individual trajectories but about the couple as a unit. Findings from these studies add evidence for the variability present in the change in relationship satisfaction over time in newlywed couples (Kanter & Proulx, 2021), in couples following a relationship education program (Kanter et al., 2023), in couples during the transition to parenthood (Stadelmann, 2022), and in long-term stable couples (Roth et al., 2025). The findings of these studies differ in terms of the numbers of subgroups identified, varying between two and three, and in terms of the identified trajectory shapes. While stability and/or varying degrees of declines are identified in all studies (Kanter et al., 2023; Kanter & Proulx, 2021; Roth et al., 2025; Stadelmann, 2022; Su et al., 2023), only three studies identified subgroups with increasing relationship satisfaction (Anderson et al., 2010; Kanter & Proulx, 2021; Roth et al., 2025). Additionally, a subgroup with an increasing relationship satisfaction trajectory was found in couples following a relationship education program (Kanter et al., 2023). Altogether, the current state of the literature suggests considerable between-couple variability in the change in relationship satisfaction across time, as opposed to the long-standing assumption of a universal decline in relationship satisfaction for all couples. Notably, previous findings on subgroup trajectories predominantly stem from newlywed samples, with the exception of the study by Anderson et al. (2010), which has focused on long-term couples. Interestingly, this is also one of the few studies that identified a subgroup with an increasing relationship satisfaction. It may therefore be the case that the positive effects of a longer relationship duration as assumed in attachment theory only become evident after a considerable period of time, which highlights the importance of studying such long-term relationships.

### 1.2. Predictors of Changes in Relationship Satisfaction

Given the identified variability in the change in relationship satisfaction (e.g., Proulx et al., 2017), it is central to identify the predictors of such variability. For example, which factors enable couples to maintain a high relationship satisfaction over time or make couples more vulnerable to decline? From a theoretical perspective, in both the Vulnerability–Stress–Adaptation model (VSA; Karney & Bradbury, 1995) and the Stress–Divorce Model embedded in the Systemic Transactional Model (Bodenmann, 2005), interpersonal behaviors play a relevant role for relationship satisfaction. In the VSA (Karney & Bradbury, 1995), interpersonal behaviors directly impact relationship satisfaction, and interpersonal processes are, in turn, impacted by enduring vulnerabilities and external stress. The Stress–Divorce Model and the Systemic Transactional Model (Bodenmann, 2005) also emphasize the importance of interpersonal behaviors for relationship satisfaction when facing external stress (i.e., dyadic coping) or internal stress (i.e., conflict communication). Looking at the empirical contributions, a great amount of research focused on examining predictors of relationship satisfaction (see Righetti et al., 2022 for a review). Besides this review, two recent studies pooled data from various datasets to examine predictors of (change in) relationship satisfaction, which both highlight the importance of relationship-specific interpersonal variables for relationship satisfaction (Joel et al., 2020; McNulty et al., 2021).

Two types of interpersonal behaviors were repeatedly shown to be important for relationship satisfaction, both cross-sectionally and longitudinally (e.g., Bodenmann et al., 2006; Falconier et al., 2015; Kanter et al., 2021; Ruffieux et al., 2014; Rusu et al., 2020): Dyadic coping (Bodenmann, 1995, 1997) and communication during conflict (M. D. Johnson et al., 2022; Woodin, 2011). Dyadic coping represents a process of how partners communicate about stress, support each other in times of stress, and jointly cope with stressors (Bodenmann, 1995, 1997). Dyadic coping is applied when facing extra-dyadic stress (stress that originates outside of the couple, e.g., work-related stress), whereas in conflict communication, typically, intra-dyadic stress (stress that originates within the couple, e.g., tensions of conflicts due to different needs or goals) is addressed (Randall & Bodenmann, 2009). These two relationship skills are relevant for several reasons: (a) stressors, be they extra- or intra-dyadic, are often encountered (Timmons et al., 2017); (b) the two skills are modifiable (Bodenmann et al., 2009) and can thus be addressed in prevention or therapy; and (c) dysfunctional communication figures among the most frequent reasons for couples to seek therapy (Doss et al., 2004).

While there is a solid ground of literature pointing to the relevance of different interpersonal variables for relationship satisfaction, predicting the actual change in relationship satisfaction posed a challenge even when using 43 pooled datasets (Joel et al., 2020). McNulty et al. (2021) recently stated that “Understanding the factors that explain declines in marital satisfaction is one of the most pressing challenges for relationship science” (p. 1). Given that subgroup analyses enable the detection of different patterns of change in the data (instead of modeling one average trajectory that might not well represent individual trajectories; Nagin, 1999, 2005), using subgroup analyses for understanding changes in relationship satisfaction might be a valuable approach. Indeed, both support and communication were found to be correlated with subgroup membership of relationship satisfaction trajectories in prior research: Lower partner support increased the likelihood of being in a group with a lower initial relationship satisfaction in couples during the transition to parenthood (Don & Mickelson, 2014), and better communication patterns increased the likelihood of being in a subgroup with higher initial relationship satisfaction in newlywed couples (Lavner & Bradbury, 2010). Expanding this line of research to couples in long-term relationships, including both dyadic coping and communication, is an important next step for understanding their relevance.

### 1.3. Current Study

Building upon previous findings in Roth et al. (2025), we aimed to investigate predictors of the identified relationship satisfaction subgroup trajectories in long-term stable couples. In Roth et al. (2025), using dyadic latent class growth analysis, three subgroups of change were identified: Class 1 (C1) represents a *high relatively stable* subgroup encompassing 65% of couples who reported high initial satisfaction with stability in men’s and slight decreases in women’s relationship satisfaction, Class 2 (C2) represents a *high declining* subgroup consisting of 19% of couples who reported high initial relationship satisfaction but large declines over time, and Class 3 (C3) represents a *low increasing* subgroup with 17% of the couples who reported the lowest level of initial relationship satisfaction among the three subgroups but a slight increase in relationship satisfaction over time (Roth et al., 2025). The estimates found in Roth et al. (2025) are presented in Table 1.

As theoretical and empirical contributions highlight the importance of interpersonal behavioral processes, we examined dyadic coping and communication during conflict as two types of relationship skills that have repeatedly been associated with relationship satisfaction (Falconier et al., 2015; Kanter et al., 2021). We expected these relationship skills measured at the first time point to distinguish between subgroup trajectories. Specifically, we hypothesized that higher dyadic coping and communication skills (i.e., more positive and less negative dyadic coping and communication, respectively) would be associated with subgroups that report higher initial relationship satisfaction and show stability or less steep declines over time. Thus, the *high relatively stable* subgroup and the *high declining* subgroup were expected to be distinguished by their relationship skills at baseline, with the *high relatively stable* subgroup reporting better relationship skills. The least beneficial levels of dyadic coping and communication (less positive and more negative dyadic coping and communication, respectively) at baseline were expected to be associated with the *low increasing* subgroup, given their already low relationship satisfaction at the beginning of the study. While newlywed couples are typically satisfied at the beginning of their relationship, this study focuses on stable, long-term couples, who—due to the assumed low level of skills—may have developed this low relationship satisfaction over time, which was observed at the beginning of the current study. In addition to baseline differences in relationship skills, we examined the extent to which the relationship skills change over time. We hypothesized stability in relationship skills to be associated with stability in relationship satisfaction (C1), a decrease in relationship skills to be associated with a decrease in relationship satisfaction (C2), and an increase in relationship skills to be associated with an increase in relationship satisfaction (C3).

## 2. Materials and Methods

### 2.1. Transparency and Openness

The current study was not preregistered, and the data are not publicly accessible. Material and results for the identification of subgroup trajectories can be found in Roth et al. (2025).

### 2.2. Participants and Procedure

We used data from a longitudinal research project where 368 mixed-gender couples (*n* = 736 individuals) in annual assessments (T1–T10) over 10 years (2011–2021) were followed. Couples were recruited via newspaper and radio announcements. To be eligible for inclusion, couples had to be in their current relationship for at least one year, fluent in German, and at least 18 years old. The Ethics Committee of the University of Zurich approved the study (No. 17.8.2; 19.8.13; 20.6.18; 24.08.27), and all participants gave their written informed consent. At each assessment, participants filled in questionnaires on a wide range of individual and dyadic variables (see https://doi.org/10.15139/S3/IUGVBK for more information on the study). For the current analyses, questionnaire data from all ten measurement time points were used. The final analytic sample used in Roth et al. (2025) consisted of 300 mixed-gender couples (*n* = 600 individuals at T1). The attrition rate was 51%, leading to 189 couples dropping out throughout the study (including separations). We excluded couples that separated during the study (*n* = 68 couples), as missing data of these couples cannot be assumed to be missing at random. Furthermore, we were interested in examining long-term stable couples and therefore focused on couples that remained in their relationship throughout the study. For the included couples, missing data was handled using full information maximum likelihood as the Little Test (Little, 1988) supported the assumption of missing at random (see Roth et al., 2025 for more details). The current analyses build upon these findings and therefore use the same final analytic sample. At T1, women were, on average, *M* = 51.1 years old (*SD* = 17.8 years), and men were, on average, *M* = 53.0 years old (*SD* = 17.7 years). At baseline, relationship duration was, on average, *M* = 25.1 years (*SD* = 18.3 years), and 75% (*n* = 224) of couples were married, with 88% of these in their first marriage and 12% in their second marriage. Seventy-two percent of women and 71% of men reported having at least one child. Most participants were Swiss (86% of women, 85% of men) or German (6% of women, 8% of men). The sample was highly educated, with 29% of women and 51% of men holding a university degree and had middle to higher socioeconomic backgrounds (median income of 120,000 Swiss Francs per couple, which is approximately 140,000 US Dollars).

### 2.3. Measures

Dyadic Coping (DC): We used the validated original German version of the Dyadic Coping Inventory (DCI; Bodenman, 2008) to assess DC. The DCI consists of 37 items and assesses each partner’s report of own (e.g., “I show empathy and understanding to my partner”) and the partner’s DC (e.g., “My partner shows empathy and understanding to me”) with daily stressors. We used the perceived partner’s positive DC (5 items) and negative DC (4 items). Items are answered on a 5-point scale indicating the frequency of the respective DC (1 = *never*/*very rarely* to 5 = *very often*). Reliability was good for positive DC for women (α = 0.82) and men (α = 0.80), respectively, and questionable α = 0.68 (men) to acceptable α = 0.75 (women) for negative DC in the current study.

Communication: To measure communication during conflict resolution, we used the 12-item short version of the original German Marital Communication Questionnaire (MCQ; Bodenmann, 2000; Bodenmann et al., 2009), which is based on Gottman’s affective communication categories (Gottman, 1994). Four items cover positive communication (e.g., listening, trying to understand the partner) and eight items cover negative communication (e.g., deny responsibility, blame the partner). Both partners rated their own, as well as their partner’s, communication behavior. Positive and negative communication perceived by partner subscales were used for the current analyses. Items are answered on a 6-point scale ranging from 1 = *never* to 6 = *always*. The validity of the MCQ has been documented (Bodenmann, 2000) and the reliability in the current study proved to be acceptable α = 0.79 (men) to good α = 0.83 (women) for positive communication and good for negative communication for women (α = 0.81) and men (α = 0.80), respectively.

### 2.4. Data Analyses

The current analyses were based on Dyadic Latent Class Growth Analyses (dyadic LCGA), in which the authors identified latent classes with distinct trajectories of relationship satisfaction over time (for details, see Roth et al., 2025). The final solution consisted of three latent subgroups with distinct relationship satisfaction trajectories over the 10-year timespan of the study, with relationship duration in years as the only control variable in the model (Roth et al., 2025). In the current study, we included DC and communication subscales, assessed at T1, as predictors of latent classes. Additionally, we tested whether a T1-T10 difference score in relationship skills differed between subgroups. To do so, we used a three-step approach (BCH; named after Bolck et al., 2004) to assess mean differences in both partners reports of DC and communication subscales across latent classes. To test the hypotheses regarding changes in relationship skills and their association with the subgroups, we examined how relationship skills changed over time within each subgroup. To do so, we tested the difference between T1 and T10 for significance, with a positive value indicating an increase in skills and a negative value indicating a decrease. Both for within-group changes in relationship skills over time (24 tests in total) and for between-group comparisons (48 tests in total), we applied a Bonferroni–Holm correction to account for multiple testing. Data preparation, descriptive statistics, and Bonferroni–Holm corrections were conducted in RStudio version 2023.06.1+524 (R Core Team, 2023). The dyadic LCGA, including the BCH method, was estimated in Mplus Version 8.7 (Muthén & Muthén, 1998–2017).

## 3. Results

Means, standard deviations, and correlations of all study variables are presented in the Supplementary Material (Table S1), and Table 2 depicts a condensed version of the table, including the predictors and relationship satisfaction at baseline and at the last time point. The descriptive statistics show that the sample was satisfied overall and exhibited high positive, and low negative DC and high positive and low negative communication.

**Figure 1:**
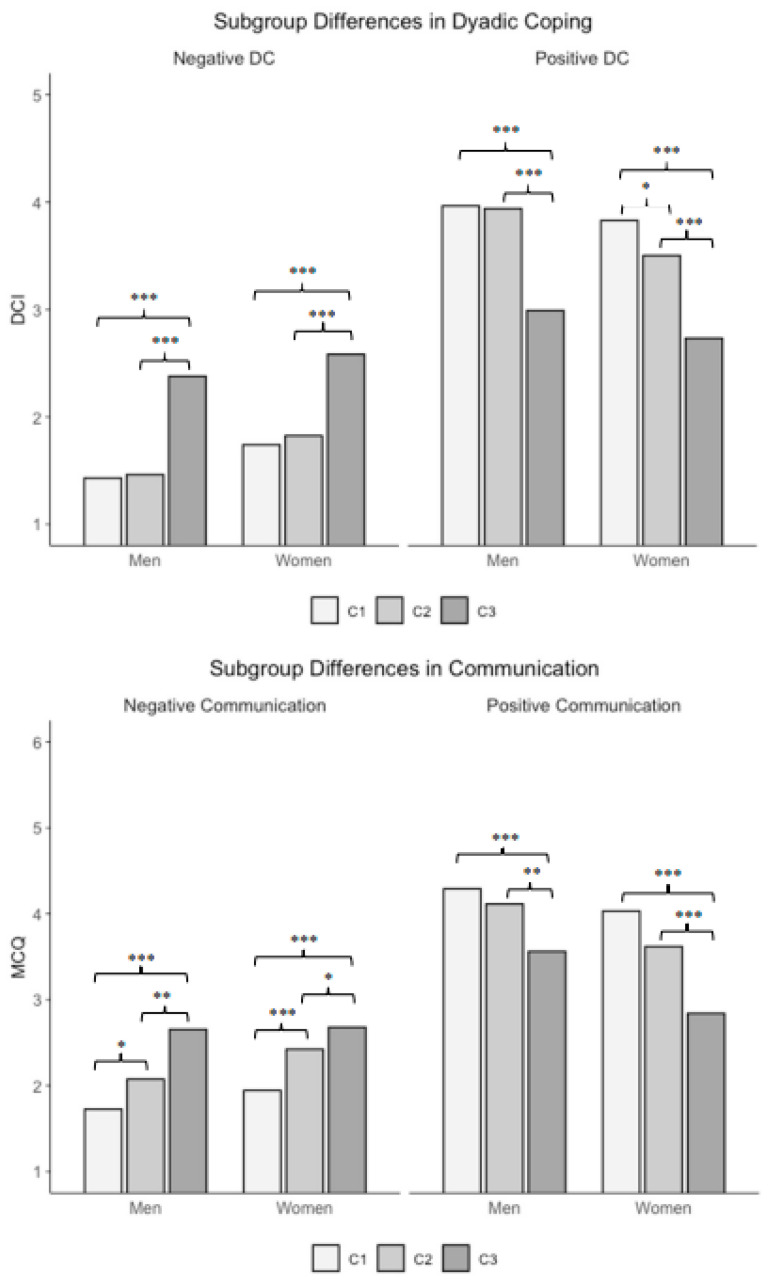
*Subgroup differences in dyadic coping and communication*. DCI = dyadic coping inventory; MCQ = marital communication questionnaire; DC = dyadic coping; * p < 0.05. ** p < 0.01. *** p < 0.001.

To assess the predictor’s relevance for relationship satisfaction subgroup trajectories, we next present the findings on between-group mean differences in dyadic coping and communication at baseline and in the difference score for women and men. Additionally, we report within-group changes over time in dyadic coping and communication for women and men. Means, results of equality tests across subgroups using the BCH method, and of within-group change are depicted in Table 3. The mean differences at baseline are graphically represented in Figure 1.

### 3.1. Positive and Negative Dyadic Coping

Although women in the *high relatively stable* subgroup descriptively reported the highest levels of positive DC at baseline, equality tests showed that women in the *high relatively stable* and in the *high declining* subgroup perceived similar amounts of positive DC by their partner, and those were significantly higher than the perceived positive DC in the *low increasing* subgroup (C1 = C2 > C3). The same pattern was found in men (C1 = C2 > C3). Tests for within-group change showed that both women and men in the *high declining* subgroup reported a significant decrease in perceived positive DC, whereas women and men in the *high relatively stable* and the *low increasing* subgroup showed no significant change and did not significantly differ from each other in their perceived positive DC change.

For negative DC at baseline, equality tests showed the same pattern of results as for positive DC for both women and men: both subgroups with high initial relationship satisfaction (the *high relatively stable* and the *high declining* subgroup) reported similar levels of negative DC, and those were lower than the perceived negative DC in the *low increasing* subgroup (C1 = C2 < C3). Tests for within-group change in negative dyadic coping showed that women and men in the *high declining* subgroup significantly increased in their perceived negative DC, while the *low increasing* subgroup significantly decreased in their perceived negative DC.

While we assumed baseline differences in positive and negative DC between the *high relatively stable* and the *high declining* subgroup, with more beneficial levels of relationship skills for the former, contrary to our hypothesis, we found no such baseline differences. In line with our hypothesis, the *low increasing* subgroup reported the least beneficial levels of DC (lower positive, higher negative) at baseline. Regarding the change in dyadic coping, in line with our hypothesis, we found stability in positive and negative DC for both women and men in the *high relatively stable* subgroup. In the *high declining* subgroup, a decrease in positive DC with a simultaneous increase in negative DC was present for both women and men, thereby confirming our hypothesis. Lastly, our hypothesis about an improvement in DC in the *low increasing* subgroup was partly confirmed: while both women and men reported a significant decrease in negative DC, neither of them reported an increase in positive DC.

### 3.2. Positive and Negative Communication

For positive communication, although women in the *high relatively stable* subgroup reported the highest levels of perceived positive communication at baseline, equality tests showed that the *high relatively stable* and the *high declining* subgroups did not significantly differ. Women in the *low increasing* subgroup reported the lowest levels of perceived positive communication at baseline (C1 = C2 > C3). The same pattern was found for men (C1 = C2 > C3). Tests of within-group change revealed that both women and men in the *high relatively stable* subgroup reported a significant decrease in positive communication, while the decrease was not significant in the *high declining* and *low increasing* subgroups.

We found that negative communication perceived by women at baseline was significantly lower in the *high relatively stable* subgroup than in the *high declining* and the *low increasing* subgroups. The latter two subgroups did not significantly differ in the negative communication reported by women (C1 < C2 = C3). A different pattern of results was found for negative communication perceived by men: At baseline, no significant differences were found between the *high relatively stable* and the *high decreasing* subgroups, and both subgroups reported lower negative communication than the *low increasing* subgroup (C1 = C2 < C3). None of the subgroups reported a significant change in negative communication.

Consistent with our hypothesis, we found negative communication at baseline to distinguish between the *high relatively stable* and the *high declining* subgroups, with the latter reporting more negative communication at baseline. Contrary to our hypothesis, negative communication in men and positive communication (women and men) at baseline did not distinguish between the *high relatively stable* and the *high declining* subgroup in either men or women. In line with our hypothesis, we found the lowest positive communication (women and men) and the highest negative communication (men) in the *low increasing* subgroup at baseline. We found no evidence to support our hypothesis regarding a change in positive or negative communication in either women or men. Results showed stability in those variables within all subgroups, except for a significant decrease in positive communication (women) in the *high relatively stable* subgroup.

## 4. Discussion

Although relationship satisfaction has been widely studied, comparatively less is known about patterns of change in relationship satisfaction and its predictors in long-term stable relationships. Therefore, this study built upon previously identified subgroup trajectories reported in Roth et al. (2025) and examined predictors of subgroup trajectories. We expected relationship skills measured at the first time point to distinguish between subgroup trajectories. Furthermore, we expected the within-group change in relationship skills across time to explain patterns of change in relationship satisfaction. In sum, we found significant differences between classes at baseline as well as within-group changes consistent with our hypotheses, which underline the importance of dyadic skills and their enhancement (Bodenmann et al., 2009) for long-term relationship well-being.

Given the identified subgroup trajectories in Roth et al. (2025), the key question is why two subgroups (C1 and C2), both with high relationship satisfaction at the start of the study, show such distinct patterns over time, with one group staying relatively stable and one group declining strongly? We hypothesized that both baseline levels and changes in dyadic coping and communication during conflict would play a relevant role in answering this question. Contrary to our expectations, these two subgroups were similar in most relationship skills at baseline, with the exception of negative communication in women, which was significantly higher in the *high declining* than the *high relatively stable* subgroup. Thus, couples that manage to communicate functionally (i.e., with less negative communication as perceived by women) when dealing with intra-dyadic stress appear better protected from deterioration of their relationship, while the other relationship skills at baseline did not sufficiently distinguish the two subgroups. However, the change in relationship skills over time—particularly in dyadic coping—seems promising in explaining the stability vs. decline in relationship satisfaction. While stability in relationship skills was found in the subgroup with stability in relationship satisfaction, a deterioration in relationship skills with increasing negative and decreasing positive DC was present in the *high declining* subgroup, making them more vulnerable to a decrease in relationship satisfaction.

In line with our hypothesis, the least favorable baseline levels of dyadic coping and communication were found in the *low increasing* subgroup. This aligns with meta-analytic findings, showing that lower positive and higher negative dyadic coping are associated with lower relationship satisfaction (Falconier et al., 2015). Furthermore, in previous studies using group-based approaches, more negative communication patterns (Lavner & Bradbury, 2010) and lower partner support (Don & Mickelson, 2014) were associated with subgroups with lower initial relationship satisfaction in newlyweds and in couples during the transition to parenthood, respectively. Our findings, therefore, extend this pattern of results to long-term couples.

However, despite the low levels of relationship skills at the beginning of the study, these couples were able to increase their relationship satisfaction over the course of the study. While most studies did not document increasing relationship satisfaction in observational studies without intervention (see Proulx et al., 2017 for a review), some studies did identify subgroups with increasing relationship satisfaction (Anderson et al., 2010; Kanter & Proulx, 2021). Further studies are needed to potentially replicate such a pattern, to explore the conditions under which it may occur, and to examine the reasons for the increase in relationship satisfaction. Meta-analytic results on relationship satisfaction across the lifespan typically point to a decrease during young adulthood and the early years of a relationship. After reaching a low point around ten years, relationship satisfaction tends to rise again until about 20 years of relationship duration (Bühler et al., 2021). It is thus possible that couples in the *low increasing* subgroup fall in this developmental time period of increasing relationship satisfaction; this would align with the finding that couples with a longer relationship duration were significantly more likely to belong to the *low increasing* subgroup than to the *high declining* subgroup (Roth et al., 2025). However, the question remains about what enables those couples to increase their relationship satisfaction. Our results on the change in relationship skills point to the importance of negative DC for explaining the increase in relationship satisfaction. Both women and men in the *low increasing* subgroup reported a decrease in negative DC, while the change in the other relationship skills was not significant. Thus, reducing negativity when supporting a partner in coping with stress appears to be particularly important for restoring low relationship satisfaction. In addition to the relationship skills examined in the present study, other factors may also play a relevant role in promoting positive changes in relationship satisfaction. For example, and in line with the later stages of the relationship of the *low increasing* subgroup, it is possible that the couples within this subgroup experienced decreasing levels of stress and conflict over the course of the study, since they might have already overcome stressors, such as the transition to parenthood, that are known to impact relationship satisfaction (Don & Mickelson, 2014). This remains to be further investigated in future research.

Altogether, our results suggest that both for distinguishing the two subgroups with high initial relationship satisfaction but strong declines within one subgroup as well as for explaining the increase in relationship satisfaction in a third subgroup, dyadic coping seems to be particularly important. This is further reinforced by the unexpected finding that the high relatively stable subgroup reported a decrease in positive communication, yet continued to exhibit consistently high relationship satisfaction, raising questions about the relevance of communication during conflict for changes in satisfaction. This finding warrants replication in future studies. Importantly, conflict frequency and intensity may be critical factors to consider, as conflict communication is likely more relevant for high-conflict couples than for generally satisfied couples, such as those in the present study.

While exploring gender differences was not the focus of this study, the overall pattern of results was similar for women and men. However, across relationship skills, women generally perceived less favorable baseline levels and patterns of change in their partner’s skills compared to men. Lastly, it will also be important to gain a better understanding of how couples manage to improve their relationship skills, as was observed in part of the present study, without a skills-based intervention. In the current study, some couples reported that participating in the study prompted them to reflect more on their relationship, which might have served as some form of low-key intervention.

Several limitations need to be addressed when interpreting the results. The present findings are from a sample with relatively high socioeconomic status and relationship satisfaction, whereby all couples remained above the threshold for relationship distress even after 10 years, as well as high positive and low negative communication and DC. Furthermore, we excluded couples who separated during the study. While our goal was to specifically examine couples in long-term stable relationships, this exclusion also most likely means that couples with lower or more rapidly declining relationship satisfaction were omitted, potentially resulting in an overly optimistic picture of the trajectory of relationship satisfaction. Altogether, this limits the generalizability of the findings from the current study and calls for more diverse samples in terms of levels of satisfaction and relationship skills, as well as in terms of socioeconomic status, sexual orientation, gender identity, and cultural background, and their interplay are needed, as also emphasized in a recent scoping review by Randall et al. (2022). This is particularly important since dyadic processes might operate differently in lower-income couples or couples in more diverse environments (Karney & Bradbury, 2020). For example, contrary to expectations, the impact of relationship education programs on well-being and relationship stability was negligible in lower-income couples (Wood et al., 2014). Potentially, this might also be due to different challenges lower vs. higher income couples face, whereby lower income couples were more likely to report finances or substance abuse (Trail & Karney, 2012). Thus, while dyadic coping seemed to be more important in explaining relationship satisfaction trajectories than communication in the current sample, replicating previous findings (Nussbeck et al., 2012), communication during conflict might be more important in high-conflictual couples and according to the severity of the conflict issue (McNulty & Overall, 2025). Here as well, it is important to consider more diverse samples, as prior research indicates that both the sociocultural context and gender influence aggressive behavior during conflicts as well as the associated negative emotional experiences (Sowan-Basheer, 2020). Moreover, while our use of Dyadic Latent Class Growth Analyses to identify groups yielded results that are largely commensurate with groups identified in prior studies, alternative methods such as Growth Mixture Modeling have been shown to capture more within-person variability in satisfaction trajectories (Joiner et al., 2024). Importantly, we used a relatively simple measure of change in relationship skills by calculating a difference score. This approach did not allow us to examine potentially meaningful bivariate associations over time between relationship skills and relationship satisfaction. Such analyses are not feasible with the relatively small class sizes resulting from the current Dyadic Latent Class Growth approach but should be addressed in future research using alternative statistical methods, such as Random-Intercept Cross-Lagged Panel Models. Lastly, several limitations pertain to the used measures: First, all variables were assessed using self-report measures, which may be subject to common method bias and social desirability, whereas, in contrast, assessing communication and DC through behavioral observation would allow for more objective measures and for analyzing temporal dynamics. Second, while the Dyadic Coping Inventory (Bodenmann, 2008) is a validated measure with international replication, the reliability of the negative dyadic coping subscale was unusually low (α = 0.68) for men in the current sample. This is likely due to the overall small number of four items in the subscale, alongside a floor effect with relatively low variance in the current sample. Results on negative dyadic coping in men should therefore be interpreted with caution. Third, because the context and problem intensity of the conflict were not assessed, we could not consider and build on the interesting finding by McNulty and Russell (2010), suggesting that negative communication can be beneficial in cases of high problem intensity.

Future research should continue to investigate relationship skills within group-based trajectories of relationship satisfaction to enhance a more detailed understanding of patterns of relationship skills and their relevance for different trajectories in relationship satisfaction. In line with this, future research might additionally benefit from studying relationship skills repeatedly over time to gain a deeper understanding of how these skills develop and co-evolve with relationship satisfaction over time. Additional predictors should be incorporated in those considerations, whereby stress is likely to be a relevant variable. Furthermore, positive interpersonal processes, where positive emotions are the core of the interaction, such as gratitude or capitalization (sharing good news) (Algoe, 2019) might reveal interesting additional insights for understanding couples’ long-term relationship well-being.

## 5. Conclusions

Taken together, our findings point to the relevance of relationship skills for the understanding of different relationship satisfaction trajectories in long-term stable couples. Importantly, if aiming to predict change in relationship satisfaction, it should be adequately modeled in the first place. Using Dyadic Latent Class Growth Analyses, which allows us to model different patterns of change instead of an overall trajectory over the whole sample, seems valuable in this regard. Our findings indicate that subgroups of relationship satisfaction trajectories can be systematically differentiated based on their relationship skills. Highly satisfied and relatively stable couples can be distinguished from satisfied but strongly declining couples mainly in terms of higher negative communication during conflict (perceived by women) at baseline and by a deterioration in dyadic coping (increase in negative and decrease in positive DC) in the latter couples. This highlights the importance of putting emphasis on lowering negative and increasing positive dimensions simultaneously in couples’ interactions. Couples with the lowest relationship satisfaction showed the least beneficial levels of communication and dyadic coping, requiring strengthening these couples across different dimensions of interpersonal interactions. A promising approach, particularly because improvements in skills in these couples were associated with improvements in relationship satisfaction.

The more detailed understanding gained from the current study, regarding which relationship skills might be more influential for which couples, holds important implications for professionals working with couples: First, this knowledge can be used to inform couples through psychoeducation about different relationship trajectories and the importance of relationship skills to prevent declines in relationship satisfaction but also to enable improvements in relationship satisfaction through improvements in relationship skills. Second, relationship education programs, such as the Prevention and Relationship Education Program (PREP; Allen et al., 2015) that aims to maintain functional communication patterns or the Couples Coping Enhancement Training (CCET; Bodenmann & Shantinath, 2004) that aims to strengthen dyadic coping skills, are of relevance to prevent declines in relationship satisfaction over time or to enable improvements in relationship satisfaction (cf. Rogge et al., 2013; Williamson et al., 2016). According to our results, particularly strengthening couples in their dyadic coping might be valuable in the long term (see CCET, Bodenmann & Shantinath, 2004). Importantly, based on our findings, rather than relying on a single skills-training session, couples should be encouraged and provided with opportunities to continuously nurture their relationship through regular, repeated skills practice, since baseline measures of relationship skills only partially explained patterns of change in relationship satisfaction. Intervention formats could be adapted accordingly, emphasizing ongoing training facilitated by practitioners to better support couples in sustaining or improving relationship skills. In the future, examining relationship satisfaction and relationship skills bivariately over time and considering additional variables—such as stress or further positive interpersonal processes—rather than studying different factors in isolation, may further enrich our understanding of how relationships change over time.

## Figures and Tables

**Table 1:** Results of the dyadic latent class growth analysis with three latent classes published in Roth et al. (2025).

	Subgroups		
Model Parameter	C1: *High Relatively Stable* *n* = 194 (65%)	C2: *High Declining* *n* = 56 (19%)	C3: *Low Increasing* *n* = 50 (17%)
Intercept women	5.463 *** (0.125 ***)	5.170 *** (0.414 ***)	4.378 *** (0.551 ***)
Slope women	−0.022 ***	−0.144 ***	0.032 *
Intercept men	5.475 *** (0.114 ***)	5.232 *** (0.261 ***)	4.304 *** (0.382 **)
Slope men	−0.013	−0.121 ***	0.044 **

*Note.* Table adapted from “Positive Outcomes of Long-Term Relationship Satisfaction Trajectories in Stable Romantic Couples: A 10-Year Longitudinal Study” by Roth et al. (2025), *International Journal of Applied Positive Psychology*, *10*(8), p. 10 (https://doi.org/10.1007/s41042-024-00201-1). Licensed under CC BY 4.0 (https://creativecommons.org/licenses/by/4.0/). Details on model specification are reported in Roth et al. (2025). Values in brackets are variances of the respective estimate. C1 = Class 1; C2 = Class 2; C3 = Class 3. * *p* < 0.05. ** *p* < 0.01. *** *p* < 0.001.

**Table 2:** Means, standard deviations, and correlations of study variables.

		Women	Men						
Variable		*M* (*SD*)	*M* (*SD*)	1	2	3	4	5	6
1.	Rel Sat T1	5.20 (0.67)	5.20 (0.69)	0.56	0.49	0.55	−0.48	0.47	−0.47
2.	Rel Sat T10	4.90 (0.91)	5.10 (0.74)	0.47	0.59	0.34	−0.27	0.32	−0.33
3.	Pos DC T1	3.59 (0.78)	3.80 (0.66)	0.54	0.23	0.31	−0.58	0.66	−0.46
4.	Neg DC T1	1.90 (0.77)	1.59 (0.60)	−0.51	−0.34	−0.48	0.36	−0.47	0.53
5.	Pos COM T1	3.75 (0.94)	4.16 (0.84)	0.37	0.33	0.52	−0.38	0.21	−0.50
6.	Neg COM T1	2.15 (0.65)	1.91 (0.61)	−0.51	−0.27	−0.45	0.54	−0.45	0.51

*Note.* Values above the diagonal are for women, those below the diagonal for men; values on the diagonal are between-partner correlations; Rel Sat = relationship satisfaction; DC = dyadic coping index; COM = communication; Pos = positive; Neg = negative; T1 baseline; T10: last time point. For readability, asterisks are not depicted; all correlations are significant at *p* < 0.01.

**Table 3:** Subgroup differences and within-group change in positive and negative dyadic coping and positive and negative communication.

	Positive DC	Positive DC Δ	Negative DC	Negative DC Δ	Positive COM	Positive COM Δ	Negative COM	Negative COM Δ
Women *M* (*SE*)								
C1	3.829 (0.057)	−0.041 (0.085) *p* = 1.00	1.739 (0.059)	−0.105 (0.073) *p* = 0.52	3.995 (0.072)	−0.835 (0.160) ***p* < 0.001**	1.929 (0.047)	0.037 (0.088) *p* = 1.00
C2	3.503 (0.123)	−0.769 (0.156) ***p* < 0.001**	1.824 (0.120)	0.596 (0.146) ***p* < 0.001**	3.706 (0.152)	−0.684 (0.284) *p* = 0.10	2.378 (0.107)	0.298 (0.225) *p* = 0.56
C3	2.732 (0.137)	0.381 (0.196) *p* = 0.23	2.582 (0.130)	−0.662 (0.173) ***p* < 0.001**	2.827 (0.144)	−0.245 (0.298) *p* = 1.00	2.755 (0.118)	−0.583 (0.219) *p* = 0.06
Equality	C1 vs. C2 *p* = 0.22	C1 vs. C2 ***p* < 0.001**	C1 vs. C2 *p* = 1.00	C1 vs. C2 ***p* < 0.001**	C1 vs. C2 *p* = 0.72	C1 vs. C2 *p* = 1.00	C1 vs. C2 ***p* < 0.001**	C1 vs. C2 *p* = 1.00
tests	C1 vs. C3 ***p* < 0.001**	C1 vs. C3 *p* = 0.46	C1 vs. C3 ***p* < 0.001**	C1 vs. C3 *p* = 0.06	C1 vs. C3 ***p* < 0.001**	C1 vs. C3 *p* = 0.68	C1 vs. C3 ***p* < 0.001**	C1 vs. C3 *p* = 0.13
	C2 vs. C3 ***p* < 0.001**	C2 vs. C3 ***p* < 0.001**	C2 vs. C3 ***p* < 0.001**	C2 vs. C3 ***p* < 0.001**	C2 vs. C3 ***p* < 0.001**	C2 vs. C3 *p* = 1.00	C2 vs. C3 *p* = 0.21	C2 vs. C3 *p* = 0.07
Men *M (SE)*								
C1	3.965 (0.048)	0.038 (0.077) *p* = 0.21	1.429 (0.042)	0.034 (0.055) *p* = 1.0	4.330 (0.067)	−0.920 (0.180) ***p* < 0.001**	1.698 (0.039)	0.210 (0.079) *p* = 0.06
C2	3.940 (0.083)	−0.481 (0.124) ***p* < 0.001**	1.461 (0.079)	0.385 (0.141) ***p* = 0.05**	4.101 (0.136)	−0.642 (0.326) *p* = 0.23	1.979 (0.101)	0.471 (0.203) *p* = 0.12
C3	2.990 (0.136)	−0.117 (0.152) *p* = 1.00	2.378 (0.114)	−0.417 (0.147) ***p* = 0.05**	3.562 (0.121)	−0.793 (0.309) *p* = 0.07	2.683 (0.141)	−0.463 (0.224) *p* = 0.21
Equality	C1 vs. C2 *p* = 1.00	C1 vs. C2 *p* = 0.001	C1 vs. C2 *p* = 1.00	C1 vs. C2 *p* = 0.26	C1 vs. C2 *p* = 0.95	C1 vs. C2 *p* = 1.00	C1 vs. C2 *p* = 0.14	C1 vs. C2 *p* = 1.00
tests	C1 vs. C3 ***p* < 0.001**	C1 vs. C3 *p* = 1.00	C1 vs. C3 ***p* < 0.001**	C1 vs. C3 *p* = 0.07	C1 vs. C3 ***p* < 0.001**	C1 vs. C3 *p* = 1.00	C1 vs. C3 ***p* < 0.001**	C1 vs. C3 *p* = 0.08
	C2 vs. C3 ***p* < 0.001**	C2 vs. C3 *p* = 0.54	C2 vs. C3 ***p* < 0.001**	C2 vs. C3 ***p* < 0.001**	C2 vs. C3 ***p* = 0.05**	C2 vs. C3 *p* = 1.00	C2 vs. C3 ***p* < 0.001**	C2 vs. C3 ***p* = 0.04**

Note. C1 = high relatively stable subgroup; C2 = high declining subgroup; C3 = low increasing subgroup; Δ = T1–T10 difference; significant *p*-values are printed in bold.

## Data Availability

The data presented in this study are available on reasonable request from the corresponding author due to the data are not publicly available (due to privacy of participants). Material and results on the identification of subgroup trajectories presented in this study are available in reference Roth et al. (2025).

## Supplementary Materials

The following supporting information can be downloaded at https://www.mdpi.com/article/10.3390/bs15101361/s1: Table S1: Means, standard deviations, and correlations of study variables.

## Abbreviations

The following abbreviations are used in this manuscript:

COM	Communication
DC	Dyadic Coping
DCI	Dyadic Coping Inventory
MCQ	Marital Communication Questionnaire
