# Supplementary material for: Dyadic Coping and Communication as Predictors of 10-Year Relationship Satisfaction Subgroup Trajectories in Stable Romantic Couples
Source: Behav Sci (Basel). 2025 Oct 5;15(10):1361. doi: 10.3390/bs15101361 (PMC12561850; doi:10.3390/bs15101361)
Supplement: Supplementary file 1 [file behavsci-15-01361-s001.zip › behavsci-3789979-supplementary.pdf]

Supplemental material to Dyadic Coping and Communication as Predictors of 10-Year Relationship Satisfaction Subgroup Trajectories in Stable Romantic Couples

Table S1. *Means, Standard Deviations, and Correlations of Study Variables*

	Variable	Women	Men														
		M (*SD*)	*M* (*SD*)	**1**	**2**	**3**	**4**	**5**	**6**	**7**	**8**	**9**	10	11	12	13	14
1.	Rel Sat T1	5.20 (0.67)	5.20 (0.69)	**.56**	.67	.71	.71	.67	.69	.59	.63	.60	.49	.55	-.48	.47	-.47
2.	Rel Sat T2	5.10 (0.74)	5.20 (0.66)	.77	**.53**	.70	.73	.71	.65	.66	.58	.58	.50	.46	-.39	.39	-.37
3.	Rel Sat T3	5.20 (0.77)	5.20 (0.76)	.73	.71	**.60**	.75	.67	.64	.52	.62	.61	.47	.47	-.42	.46	-.47
4.	Rel Sat T4	5.10 (0.80)	5.20 (0.67)	.70	.66	.75	**.56**	.77	.72	.65	.72	.65	.58	.43	-.37	.44	-.42
5.	Rel Sat T5	5.10 (0.86)	5.20 (0.73)	.67	.64	.73	.75	**.56**	.75	.70	.69	.71	.56	.42	-.38	.39	-.42
6.	Rel Sat T6	5.10 (0.80)	5.10 (0.71)	.62	.57	.72	.73	.74	**.55**	.78	.77	.75	.60	.45	-.36	.43	-.42
7.	Rel Sat T7	5.00 (0.85)	5.10 (0.75)	.63	.62	.69	.71	.73	.79	**.57**	.77	.70	.54	.43	-.32	.42	-.43
8.	Rel Sat T8	5.00 (0.85)	5.00 (0.79)	.49	.50	.62	.66	.70	.73	.75	**.60**	.78	.62	.42	-.33	.40	-.37
9.	Rel Sat T9	5.00 (0.87)	5.00 (0.80)	.55	.58	.64	.67	.66	.73	.76	.74	**.51**	.68	.36	-.30	.39	-.35
10.	Rel Sat T10	4.90 (0.91)	5.10 (0.74)	.47	.48	.51	.57	.57	.59	.63	.68	.71	**.59**	.34	-.27	.32	-.33
11.	Pos DC T1	3.59 (0.78)	3.80 (0.66)	.54	.43	.43	.41	.33	.36	.40	.28	.39	.23	**.31**	-.58	.66	-.46
12.	Neg DC T1	1.90 (0.77)	1.59 (0.60)	-.51	-.44	-.48	-.47	-.40	-.44	-.43	-.33	-.35	-.34	-.48	**.36**	-.47	.53
13.	Pos COM T1	3.75 (0.94)	4.16 (0.84)	.37	.39	.43	.39	.38	.44	.38	.33	.38	.33	.52	-.38	**.21**	-.50
14.	Neg COM T1	2.15 (0.65)	1.91 (0.61)	-.51	-.51	-.51	-.43	-.43	-.41	-.46	-.27	-.34	-.27	-.45	.54	-.45	**.51**

Note. Values above the diagonal are for women, those below the diagonal for men; values on the diagonal in bold are between-partner correlations; Rel Sat = relationship satisfaction; DC = dyadic coping index; COM = communication; Pos = positive; Neg = negative; T1-T10: time point. For readability asterisks are not depicted, all correlations are significant on $p < .01$.
